# Targetoid Eruption With Oral and Ocular Mucositis in a Young Woman

**DOI:** 10.1002/ccr3.73735

**Published:** 2026-11-11

**Authors:** Pitchaya Jaruvijitrattana, Weeratian Tawanwongsri, Chime Eden

**Affiliations:** ^1^ Division of Dermatology, Department of Internal Medicine Chaophya Abhaibhubejhr Hospital Prachin Buri Thailand; ^2^ Division of Dermatology, Department of Internal Medicine, School of Medicine Walailak University Nakhon Si Thammarat Thailand; ^3^ Division of Dermatology Jigme Dorji Wangchuck National Referral Hospital Thimphu Bhutan

**Keywords:** conjunctivitis, drug hypersensitivity, mucositis, mycoplasma‐induced rash and mucositis, targetoid lesions

## Abstract

Young adults presenting with fever, targetoid lesions, and severe mucositis pose a diagnostic challenge because drug‐induced severe cutaneous adverse reactions and infection‐associated mucocutaneous eruptions may appear clinically similar. Correct identification matters because misattributing the eruption to a drug can result in an unnecessary lifelong drug allergy label, and marked ocular involvement may require prompt ophthalmological care to prevent long‐term sequelae.

## Key Clinical Message

In young patients with fever, targetoid lesions, and marked oral and ocular mucositis, consider including *Mycoplasma pneumoniae* –induced rash and mucositis in the differential diagnosis. Distinguishing this infection‐triggered syndrome from Stevens–Johnson syndrome helps avoid unnecessary drug allergy labeling.

A 20‐year‐old woman presented with fever, painful oral mucositis, ocular irritation, and a nonpruritic eruption that began on both palms and arms before extending to the trunk and limbs. Examination showed targetoid lesions, hemorrhagic erosions of the lips, and bilateral conjunctivitis with conjunctival membranes. The oral cavity, including the palate, was examined, and no palatal vesicles were observed; the genital mucosa was spared (Figure 1). She also reported a mild cough, with minimal rhonchi on examination and a normal chest radiograph. A multiplex polymerase chain reaction panel for herpesviruses was negative.

**FIGURE 1:**
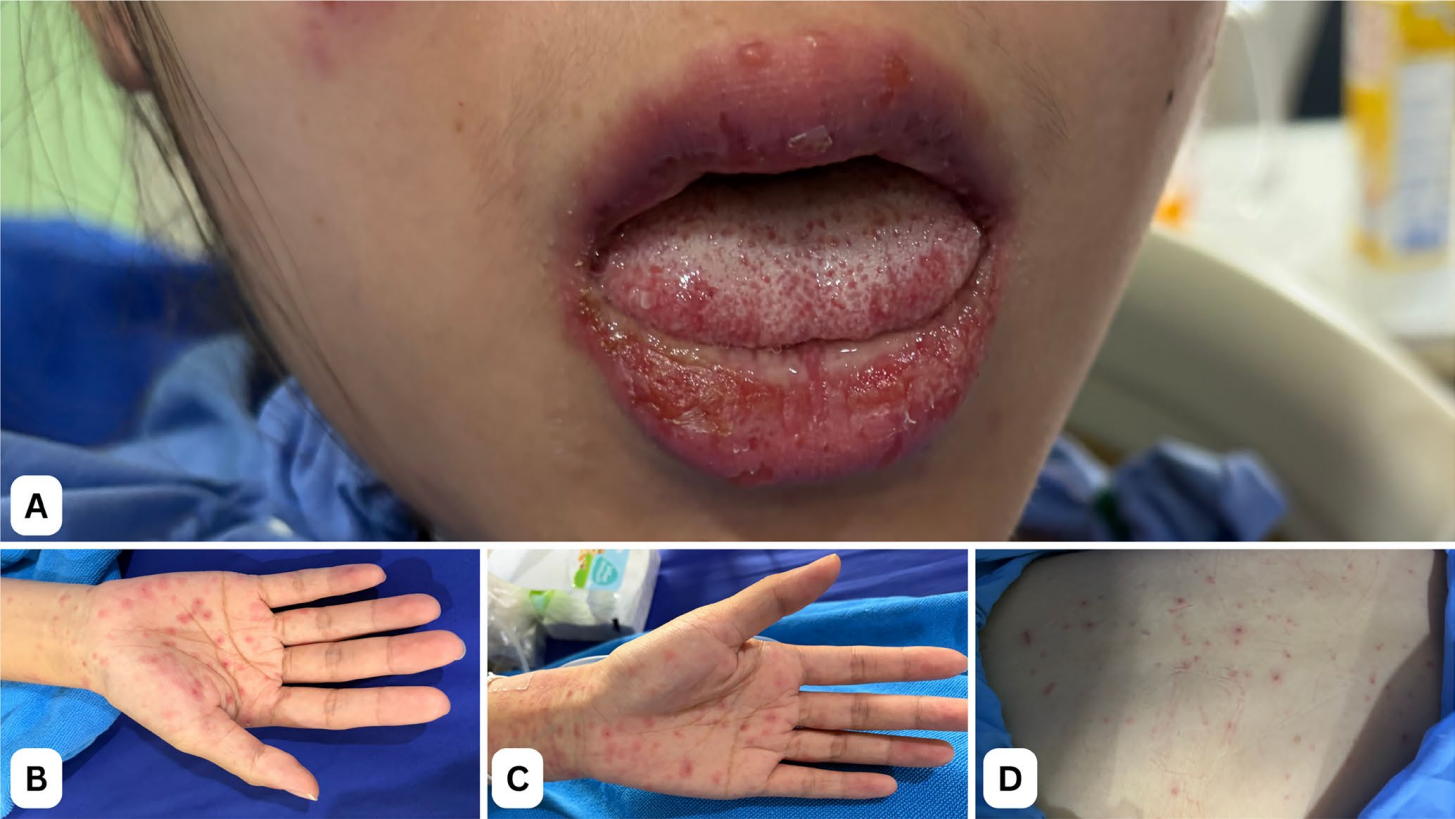
Clinical findings in the patient. (A) Hemorrhagic erosions and crusts on the lips, reflecting severe oral mucositis. (B, C) Erythematous targetoid lesions on the palms. (D) Scattered erythematous macules with small erosions on the trunk.

## 1 What is the Diagnosis?

- Stevens–Johnson syndrome
- Erythema multiforme major
-
*Mycoplasma pneumoniae* –induced rash and mucositis
- Paraneoplastic pemphigus
- Pemphigus vulgaris

## 2 Discussion

The answer is C. *Mycoplasma pneumoniae*–induced rash and mucositis.

*Mycoplasma pneumoniae* –induced rash and mucositis (MIRM) is defined by prominent mucositis affecting two or more mucosal sites, only limited epidermal detachment, and a scant eruption of vesiculobullous lesions or atypical targets in the setting of *M. pneumoniae* infection [1, 2]. In this patient, severe hemorrhagic oral erosions, bilateral conjunctivitis with conjunctival membranes, and targetoid lesions involving the palms, trunk, and extremities were consistent with this presentation. The absence of genital involvement does not exclude MIRM because oral and ocular involvement fulfill the requirement for multiple mucosal sites. The patient also had a mild cough with minimal rhonchi, although chest radiography was normal. *M. pneumoniae* IgM was subsequently positive. Respiratory PCR, rapid diagnostic testing, and paired serology were not performed.

Stevens–Johnson syndrome/toxic epidermal necrolysis (SJS/TEN) and erythema multiforme were the important alternative diagnoses. Relative to SJS/TEN, MIRM tends to affect younger patients, is triggered by infection, and shows more prominent mucositis with comparatively limited skin involvement. Erythema multiforme, by contrast, more often shows classic three‐zone target lesions with less extensive mucosal involvement [1, 2]. The patient had not taken any medications before the onset of the cutaneous and mucosal lesions. Ibuprofen and dicloxacillin were initiated only after the eruption had developed; therefore, a drug‐induced reaction was considered less likely based on the temporal relationship. Lymphocyte transformation testing and drug‐induced lymphocyte stimulation testing were not performed. Although the positive *M. pneumoniae* IgM result was compatible with the clinical presentation, IgM may persist after previous infection and therefore cannot, when used alone, confirm acute infection [2]. The diagnosis of MIRM was based primarily on the characteristic mucocutaneous presentation, concurrent mild respiratory symptoms, and the positive IgM result.

Treatment is largely supportive and includes antibiotics targeting the underlying infection; systemic corticosteroids are occasionally given for severe disease. Early ophthalmological assessment is important because conjunctival inflammation and membrane formation may lead to cicatricial complications [3]. Ophthalmology consultation was obtained. The ocular involvement was managed supportively with artificial tears. Oral azithromycin was administered for 5 days, together with artificial tears, paracetamol 500 mg orally every 6 h for pain, intravenous 5% dextrose‐normal saline, and a liquid diet. No systemic corticosteroids were administered. By the fifth day of azithromycin therapy, the fever and mucocutaneous lesions had markedly improved, with no recurrent eruption. No recurrent rash, scarring, or ocular sequelae were observed at the 1‐month follow‐up.

## 3 Conclusion

MIRM should be considered in young patients presenting with targetoid lesions and prominent oral and ocular mucositis. Early recognition helps distinguish it from drug‐induced severe cutaneous adverse reactions and supports timely multidisciplinary care.

## Author Contributions

**Pitchaya Jaruvijitrattana:** conceptualization, formal analysis, investigation, data curation, writing – original draft, writing – review and editing, visualization. **Weeratian Tawanwongsri:** methodology, validation, investigation, writing – review and editing. **Chime Eden:** writing – review and editing, supervision.

## Funding

The authors have nothing to report.

## Consent

The patient provided written informed consent for publication.

## Conflicts of Interest

The authors declare no conflicts of interest.

## Data Availability

The data that support the findings of this study are available from the corresponding author upon reasonable request.
